# The VOICES Typology of Curatorial Decisions in Narrative Collections of the Lived Experiences of Mental Health Service Use, Recovery, or Madness: Qualitative Study

**DOI:** 10.2196/16290

**Published:** 2020-09-18

**Authors:** Caroline Yeo, Laurie Hare-Duke, Stefan Rennick-Egglestone, Simon Bradstreet, Felicity Callard, Ada Hui, Joy Llewellyn-Beardsley, Eleanor Longden, Tracy McDonough, Rose McGranahan, Fiona Ng, Kristian Pollock, James Roe, Mike Slade

**Affiliations:** 1 School of Health Sciences Institute of Mental Health University of Nottingham Nottingham United Kingdom; 2 Institute of Health and Wellbeing University of Glasgow Glasgow United Kingdom; 3 School of Geographical & Earth Sciences University of Glasgow Glasgow United Kingdom; 4 Greater Manchester Mental Health NHS Foundation Trust Manchester United Kingdom; 5 Department of Psychology Mount St. Joseph University Cincinnati, OH United States; 6 Unit of Social and Community Psychiatry Queen Mary University of London London United Kingdom; 7 School of Health Sciences University of Nottingham Nottingham United Kingdom; 8 National Institute for Health Research ARC East Midlands University of Nottingham Nottingham United Kingdom

**Keywords:** personal narrative, mental health recovery, decision making

## Abstract

**Background:**

Collections of lived experience narratives are increasingly used in health research and medical practice. However, there is limited research with respect to the decision-making processes involved in curating narrative collections and the work that curators do as they build and publish collections.

**Objective:**

This study aims to develop a typology of curatorial decisions involved in curating narrative collections presenting lived experiences of mental health service use, recovery, or madness and to document approaches selected by curators in relation to identified curatorial decisions.

**Methods:**

A preliminary typology was developed by synthesizing the results of a systematic review with insights gained through an iterative consultation with an experienced curator of multiple recovery narrative collections. The preliminary typology informed the topic guide for semistructured interviews with a maximum variation sample of 30 curators from 7 different countries. All participants had the experience of curating narrative collections of the lived experiences of mental health service use, recovery, or madness. A multidisciplinary team conducted thematic analysis through constant comparison.

**Results:**

The final typology identified 6 themes, collectively referred to as VOICES, which stands for values and motivations, organization, inclusion and exclusion, control and collaboration, ethics and legal, and safety and well-being. A total of 26 subthemes related to curation decisions were identified.

**Conclusions:**

The VOICES typology identifies the key decisions to consider when curating narrative collections about the lived experiences of mental health service use, recovery, or madness. It might be used as a theoretical basis for a good practice resource to support curators in their efforts to balance the challenges and sometimes conflicting imperatives involved in collecting, organizing, and sharing narratives. Future research might seek to document the use of such a tool by curators and hence examine how best to use VOICES to support decision making.

## Introduction

### Background

Lived experience narratives are increasingly central to medical practice [1] and web-based mental health interventions [2], with a growing interest in the use of web-based mechanisms for delivering and accessing them [3]. They can be recorded and published in different ways, such as text, video, audio, art, or mixed media [4]. They can present narratives of a single author, such as *Madness Made Me* [5], or present groups of narratives edited by a curatorial team, such as *Living with Voices* [6]. Wide-scale public access to digital media hosting platforms is a relatively recent phenomenon, which now allows the presentation of a person’s narrative to a worldwide audience [4]. Generic digital media hosting services now enable recorded versions of narratives to be shared directly with recipients on a mass scale, and thousands of lived experience narratives have been uploaded and are freely available to the public.

Narratives are *representations of a real or fictitious event or series of events* [7]. Lived experience narratives can explore events such as mental health service use or experiences of mental health problems, either grounded in a biomedical understanding of mental health or conceptualized as madness, which looks more widely at psycho-socio-political issues, emotional distress, and/or spiritual emergence [8]. Lived experience narratives may or may not be classed as recovery narratives. The term recovery narrative refers to a story told by a person about their journey of recovery. This includes elements of both adversity or struggle, and of strength, success, or survival related, at least in part, to mental health problems (ie, about recovery), and which refer to events or actions over a period of time (ie, a narrative) [9]. Recovery narratives have been studied as a mechanism for understanding how recovery happens [10] and as a recovery-promoting tool with potential benefits for the narrator, such as storytelling as a route to reframing emotional distress, as a part of narrative therapy practice [11]. Recovery narratives can also offer a source of knowledge and hope for the narrators and recipients [12].

There is disagreement and debate over the use and framing of what constitutes *recovery narratives*. Recovery in the Bin, which describes itself as a critical theorist and activist collective [13], has questioned the content and use of recovery narratives in that, for example, only *successful* or *acceptable* recovery narratives are promoted in services. They call for “a broader range of Survivor narratives to be recognised, honoured, respected and promoted that include an understanding of the difficulties and struggles that people face every day when unable to ‘recover’, not just ‘successful recovery’ type stories” [13]. Recovery began as a social justice movement led by survivors and service users with the aim to reform health services and promote patient-centered care defined as "providing care that is respectful of and responsive to individual patient preferences, needs, and values and ensuring that patient values guide all clinical decisions" [14]. The movement rejected the notion of mental health diagnosis and called for a nonpathologizing and holistic treatment of mind and body, adopting a unified language of patient-centered care rather than perpetuating, through language and action, a division between physical and mental health [15]. The narratives of mental health service use, recovery, and madness have played a role in the personal and collective journeys of narrator emancipation from psychiatric judgment [16] and have been used to reshape clinical and scientific responsibilities [6], challenge conceptualizations of biomedical paradigms [17], and inform policy change [18] or as tools of resistance, opposition, collective action, and social change [19].

As narratives about recovery or madness inherently incorporate discussions of personal distress or trauma, there are challenges in the design of curatorial processes such as personal, legal, or ethical dilemmas linked to the identification of narrators or third parties. They also include broader ethical and political issues related to the representation and dissemination of distressing and difficult material, which might affect those exposed to recovery narratives and those producing them [20]. In addition, there are unresolved questions about what it means to reinscribe the identity of the service user or survivor, through the use of recovery narratives, as one closely associated with distressing events or experiences. Moreover, issues around power and control are especially relevant in curation processes owing to the existing inequalities of power, status, or assumed credibility of service users, providers, and professionals [21]. All these issues are made more complex when one considers them intersectionally and attends to gender, ethnicity, sexuality, gender identity, and disability as well as the experience of distress.

Some guidelines for the curation of recovery narratives based on experience and expert judgment have been published, for example, guidelines by the Scottish Recovery Network [22]. However, there is no research leading to a comprehensive typology of curatorial decisions for recovery or madness narratives. There is a need to outline all the critical considerations for the curation of narratives, including those that individual curators may not automatically consider without guidance. Previous guidance may be organizationally or culturally specific and may not be relevant across different contexts. A typology might support the work of future curators of recovery or madness narrative material, for example, by informing the design of a decision aid tool [23].

Initial insights into curatorial practices have been developed in a systematic review and qualitative evidence synthesis [20]. The review included 1 research publication and 22 informal documents providing evidence of curation. These were synthesized to develop a preliminary typology that described 9 curatorial issues and choices: (1) purpose of the collection, (2) audience for the collection, (3) safety (of narrators, recipients, and third parties), (4) narrative collection process, (5) narrative selection process, (6) narrative editing process, (7) narrative presentation choices, (8) ethical and legal considerations, and (9) the societal positioning of the collection. The review only included publicly available documents. Their accuracy in reflecting decisions made around curation could not be verified and therefore may not have provided full information about all curatorial decisions made because curators may not have discussed some decisions publicly.

### Aims and Objectives

The aim of this study is to extend the prior review by directly consulting expert curators about the work of curation. The objectives of this study are to develop a typology of curatorial decisions involved in curating narrative collections of the lived experiences of mental health service use, recovery, or madness and to characterize existing approaches used in relation to identified curatorial decisions.

## Methods

### Study Approvals

The study was conducted as part of the Narrative Experiences Online (NEON) Programme [24], which is a program of work investigating whether engagement with mental health recovery narratives can influence an individual’s recovery journey (ISRCTN11152837). The study was reviewed and given a favorable opinion by the Nottingham 2 Research Ethics Committee (reference 17/EM/0401) and was approved by the UK Health Research Authority. In line with the approved protocol, all participants gave verbal consent, which was recorded on a paper consent form by the researcher. The findings will inform a future trial (ISRCTN11152837).

### Participants

The inclusion criterion for participants was experience of curating 1 or more narrative collections of the lived experiences of mental health service use, recovery, or madness. Collections were considered relevant if the majority of narratives in the collection matched any of those 3 categories, or if the curator identified the collection with any of them. The meaning of the term madness was as discussed in Mad studies [25], which challenges the biomedical interpretations and language of mental health discourse. This usage has emerged through the psychiatric survivor and peer support movement.

To maximize heterogeneity, maximum variation sampling [26] was used across the dimensions of (1) the country of publication of the collection to capture country-specific social, cultural, and political contexts and (2) the presence or absence of curator lived experience of using mental health services (ie, non–mental health service user, primary care service user, secondary care service user, voluntary hospital admission, or involuntary hospital admission) to capture service user curator perspectives. Deviant case sampling was used to illuminate both unusual and typical decision-making strategies [27].

### Procedures

A semistructured interview was conducted with an expert curator. The expert curator played a lead role over 12 years in the development of an extensive recovery narrative repository containing multiple collections, the development of guidelines for narrative collection, the conduct of narrative-based research, and the development of a recovery narrative–based online intervention. Data from the interview were used to develop an initial list of curatorial decisions and approaches. This was refined through 6 consultations with the expert curator. A preliminary typology of curatorial decisions and approaches was developed by synthesizing the initial expert curator list with material presented in an earlier systematic review [20]. The preliminary typology, which had 11 themes, can be found in Multimedia Appendix 1 [1].

Semistructured interviews were then conducted with 30 other curators, either in person (n=4) or by phone (n=17), video call (n=8), or, if no other option was available, by email (n=1). Each participant was given a unique identifier. A demographics form was used to capture the curator background. The interview schedule was initially derived from the preliminary typology using open questions relating to each of the 11 themes, and then refined over the course of the interviews as new themes emerged. Participants were asked to comment on a recent collection they had curated and whether they had considered each of the 11 themes in the process of curation and what decisions they had made with respect to these. Interviews lasted 60 to 90 min and were audiotaped and transcribed using pseudonymization.

### Analysis

The analyst team consisted of 10 researchers from a range of disciplinary (survivor research, sociology, philosophy, social anthropology, digital research, organizational development, health research, and youth work) and clinical professional (mental health nursing and clinical psychology) backgrounds. A thematic analysis of transcripts by constant comparison was conducted [28] using NVivo 12 Pro (QSR International) for coding work. First, 2 analysts (CY and LHD) coded the same 6 transcripts to establish a shared understanding of the data in the form of a preliminary coding framework. This consisted of a range of superordinate themes and subthemes describing the issues considered by curators, with subthemes selected to describe specific curatorial considerations and superordinate themes selected to provide a higher-level structure. Thereafter, 3 analysts (CY, LHD, and KP) coded a further 3 transcripts to check for consistency of coding and to refine the coding framework. The remaining transcripts were then coded by CY and LHD to further develop the coding framework. The wider analyst team met every 5 to 10 transcripts to discuss newly transcribed elements and generate a set of suggested updates to interim versions of the coding framework, such as adding, splitting, or relabeling superordinate themes, or adding or splitting subthemes. Once the analysis work was complete, the coding framework was tabulated. An illustrative question was chosen to represent each subtheme, and text coded against each subtheme was examined to identify example approaches chosen by a curator. Tables are presented in the *Results* section with illustrative transcripts fragments, each accompanied by the participant identifier, for example, [#30] for the participant allocated identifier 30. We also summarize the preliminary typology and present a descriptive analysis of participant demographics.

## Results

### Preliminary Typology

The preliminary typology (Multimedia Appendix 1) offered considerations and strategies relating to 12 themes: context, purpose, curatorial team, audience, legal and copyright, collection, selection, editing, safety and well-being, presentation, language, and ordering.

### Participants

A total of 30 interviews were conducted with curators of art exhibitions (8/30, 27%) and book or online narrative collections (22/30, 73%), with collections published in 7 countries: the United Kingdom (18/30, 60%), the United States (6/30, 20%), Canada (2/30, 7%), Brazil (1/30, 3%), Hong Kong (1/30, 3%), India (1/30, 3%), and Italy (1/30, 3%). Overall, 60% (18/30) curators had used mental health services, comprising primary care (1/30, 3%), secondary care (6/30, 20%), voluntary hospitalization (8/30, 27%), and involuntary hospitalization (3/30, 10%). Table 1 presents the additional sociodemographic characteristics collected.

**Table 1:** Sociodemographic characteristics of participants (N=30).

Characteristics		Participants, n (%)	
**Gender**			
	Female		19 (63)
	Male		11 (37)
**Ethnicity**			
	White		24 (80)
	Asian		3 (10)
	Black		1 (3)
	Multiple ethnic groups		1 (3)
	Prefer to self-describe		1 (3)
**Age (years)**			
	<25		2 (7)
	25-34		4 (13)
	35-44		13 (43)
	45-54		5 (17)
	55-64		5 (17)
	>64		1 (3)
**Sexual orientation**			
	Heterosexual		22 (73)
	LGBTQ+^a^		4 (13)
	Prefer to self-describe		2 (7)
	Prefer not to say		2 (7)
**Educational level**			
	Higher degree		23 (77)
	Bachelor’s degree		7 (23)
**Employment status**			
	Full-time paid employment		18 (60)
	Part-time paid employment		5 (17)
	Self-employed		2 (6)
	Student		2 (6)
	Volunteer		2 (6)
	Retired		1 (3)
**Did the participant declare a disability?**			
	No		23 (77)
	Yes		7 (23)
**Income, £ (US $)**			
	<10,000 (<13,000)		4 (13)
	10,000-15,000 (13,000-19,500)		1 (3)
	15,001-20,000 (19,501-26,000)		3 (10)
	20,001-35,000 (26,000-45,500)		6 (20)
	35,001-50,000 (45,501-65,000)		6 (20)
	50,001-100,000 (65,001-130,000)		5 (17)
	>100,000 (>130,000)		1 (3)
	Prefer not to say		2 (6)

^a^LGBTQ+: lesbian, gay, bisexual, transgender, queer.

### The VOICES Typology of Curatorial Decisions

The final coding framework identified 6 superordinate themes relating to the process of curation: values and motivation, organization, inclusion and exclusion, control and collaboration, ethical and legal, and safety and well-being. These themes are collectively referred to as VOICES. These are listed in Textbox 1, with all identified subthemes.

**Themes and subthemes identified in the VOICES typology of curatorial decisions.**

**Values and motivation**

- Guiding values
- Purpose
- Audience
- Context

**Organization**

- Identification
- Collection process
- Presentation
- Impact and Evaluation

**Inclusion and exclusion**

- Selection and inclusion
- Exclusion
- Editing
- Language

**Control and collaboration**

- Role of curator
- Curatorial team
- Power dynamics
- Working together

**Ethics and legal**

- Anonymization
- Consent process
- Data security
- Funding
- Payments
- Copyright and ownership
- Withdrawal process

**Safety and well-being**

- Safety and well-being
- Distress
- Trigger warnings

#### Theme 1: Values and Motivations

The values and motivations theme detailed in Table 2 included participants’ guiding values (how they conducted their work) and their purposes (why they conducted their work), the audience they aimed their collection at, and the external factors that influenced how their collections were built. The purpose of building a collection could be at an individual level, for example, giving a narrator a place to tell their story, or at a collective level, such as building an evidence base for a rights movement, or both:

She contrasted writing to self-harm…So rather than carving you know, symbols on her body, she felt that writing her story was an alternative to self-harm...and you know write in a more public fashion rather than on her body because she felt that by harming herself she was actually complicit in her own abuse and subjugation, so there was a lot of social impact there.

#30

It was to start...a human rights movement so I thought okay there’s been a black rights movement, a gay rights movement, and we need a mental health rights movement.

#19

**Table 2:** Values and motivations in creating a collection.

Subthemes	Illustrative question	Identified approaches
Guiding values	What values will guide curatorial work?	Values defined collectively (eg, community-based, political, societal) Values defined organizationally (eg, by the mental health trust that has commissioned the collection) Personal values (eg, those of the curators, narrators, or publishers)
Purpose	What are the intended purposes of the collection?	Individual purposes: Generating artistic acclaim or academic success Generating personal economic gain Enhancing the personal recovery of curators, narrators, and/or recipients Collective purposes: Building a narrative collection to act as an evidence base for rights movement Building capacity among service users, survivors, and allies Facilitating emancipatory action Enabling re-evaluation of values Improving mental health and social care services from youth to old age Opening dialogue between different voices Educating or critiquing organizational, community, societal, or political aspects or structures Supporting organization, peer support, and solidarity Enabling the definitions of mental illness to be reconceptualized Enabling the voices of recovery or madness to act as agents of change
Audience	Who are the desired audiences?	Specific subgroups (eg, activists, artists, mental health workers, people with mental health issues, problems or distress, users of mental health services, their families, and/or carers, or people with a BAME^a^ ethnicity) General public Governments or political bodies
Context	What external factors influenced curation?	Economic perspectives Cultural perspectives Organizational perspectives (eg, if the collection is curated within a mental health trust) Political perspectives Societal perspectives

^a^BAME: Black, Asian, and Minority Ethnic.

#### Theme 2: Organization

The organization theme shown in Table 3 describes the practical organizational processes of building a collection and curating. These include the identification of narrators, the collection processes used to capture narratives, communication methods with the narrator, the curatorial resources required, how narratives were presented, and how they aimed to measure the impact of the collection.

**Table 3:** Organizing and presenting collections.

Subthemes	Illustrative question	Identified approaches
Identification	How to identify and recruit narrators for the collection?	Placement of advertising (eg, social media, posters, leaflets) Working with charities Working with health services Engaging with existing community groups Use of existing contacts of the curator Engaging with recovery education programs Targeting specific communities As part of a wider research or arts project
Collection process	How to capture and/or produce the narratives?	By email or post Through an interview Through one-on-one creative activities Through a video call Through group workshops (sometimes scheduled alongside other training activities)
	How to communicate with the narrator?	Email Face-to-face Phone Video call
	What resources are required?	Clinical support Funding Peer support People Time
Presentation	How will the narratives be presented?	Exhibits at an art exhibition Book Booklet Event Film Forum Online collection Song Video
Impact and evaluation	How do you measure the impact of the collection?	Analysis of feedback forms Popularity of the collection as determined by the audience size Through a research project evaluation

#### Theme 3: Inclusion and Exclusion

The inclusion and exclusion themes shown in Table 4 include the decisions relating to the selection and inclusion or exclusion of narrators or narratives and editing of narratives and language. Some participants described their power in the process of curation as they aimed to ensure that certain voices and narratives were not ignored or silenced owing to their social or political dimensions:

It is real life stories, it is truth, it is from the underground, it is the untold stories, it is for people who are on the margins of society, it is those who do not have a voice, so you know I think it is hugely important and it is giving value to their voice and you know, it is a whole world really that is not seen in the mainstream, so I think it has got huge political relevance.

#24

**Table 4:** Selection, inclusion and exclusion of narratives, narrators, and language.

Subthemes	Illustrative question	Identified approaches
Selection and inclusion	On what basis should narrators be selected for inclusion?	Clinically defined diagnosis of the narrator Demographic factors (eg, narrator is a member of a BAME^a^ community) Place of residence (eg, in a collection focused on a particular country) History of narrator service usage Narrator’s personal philosophy, values, or views
	On what basis should narratives be selected for inclusion?	Content of the narrative Curator-defined quality of writing Sociopolitical standpoint of the narrative Trajectory of the narrative (eg, narrative ends hopefully) Nature of the experience described, such as in a collection focused on presenting spiritual experiences
Exclusion	On what basis should narrators be excluded?	Conflict of personal philosophy, values, or views between the curator and the narrator Legal concerns around the narrator’s current status Presence of safeguarding concerns in relation to the narrator Sociopolitical standpoint of the narrator No basis—narrators should not be excluded
	On what basis should narratives be excluded?	Defined quality of writing Legal concerns around the content of the narrative Modality of narrative Presence of safeguarding concerns in relation to the content of the narrative Sociopolitical standpoint of the narrative Narrative length Narrative structure Narrative subject No basis—narratives should not be excluded
Editing	What characteristics will a narrative be edited for?	Content Length Language No editing Style
Language	How does language need to be changed?	Alter grammar or spelling No change Remove discriminatory language Remove expletives

^a^BAME: Black, Asian, and Minority Ethnic.

Inclusion and exclusion occurred at either the narrator or the narrative level. At the narrator level, inclusion by both the curator and the narrator could be either conscious or unconscious. Some groups may not be actively approached owing to oversight or the curator may lack time, motivation, knowledge, skills, or resources to engage with certain communities or marginalized groups. In some instances, the needs that would enable inclusion were not met owing to a number of factors. These factors included a lack of funding for the narrator’s payment, not providing sufficient reasonable adjustments for people with physical or mental health disabilities, such as British sign language interpreters or wheelchair-friendly venues, or owing to misunderstanding around benefits situations. Some potential narrators self-excluded owing to the fear of different kinds of reactions, including punitive actions:

…because of the benefits situation, they preferred just to have their travel expenses and food reimbursed and things like that...there was so much fear associated with benefits and in claiming benefits that it’s just not worth it...there’s one person that wouldn’t take part because they thought that if somebody saw it they would think oh well you are well enough to be in that video and tell your story so therefore you are fit for work.

#25

At the narrative level, the inclusion and exclusion of both content and style, including vocabulary language and grammar, raised issues around maintaining the narrator’s voice and meaning and ensuring the ethical representation of cultures in which narrators were embedded. Standardization of language and grammar may be seen as exerting class power or diminishing certain communities:

We have a made up word in here and I had a lot of wrangling with the publisher because he didn’t want me to use a made up word and I was fairly insistent that the made up word said more than any real word ever could...It was her word. It was hers, it was her way of expressing herself in a story that is full of really long words actually and a really expressive story.

#16

There were issues around some people’s language as their first language was not English, I corrected their English and made it grammatically correct. I don’t think I captured that person’s voice.

#01

#### Theme 4: Control and Collaboration

It's important people have their own voice not their voice filtered through my voice.

#02

The control and collaboration themes shown in Table 5 include the types of roles adopted by participants in the curatorial process, the desired skills and experience of the curatorial team, how issues of power were considered in the process, and how different parties worked together. Participants described how they approached the role of the curator either in a distant editorial role with very limited contact with narrators, a controlling role, or in a collaborative role of varying degrees.

**Table 5:** Control and collaboration in the curation of collections.

Subthemes	Illustrative question	Identified approaches
Role of the curator	What type of role should be adopted by the curators?	Artist Coproducer Editor Facilitator Guide Inspiration Interviewer Leader Partner Peer Supporter Writer
Curatorial team	What skills and experience are needed on the curatorial team?	Academic Activism Community engagement Curatorial experience Experience as an artist Lived experience of the subject of the collection Mental health professional training
Power dynamics	How will issues of power within the curatorial team and between the curator and the narrator be addressed?	Coproduced guidelines Curator-produced guidelines Individual discussion Group discussion Not addressed or considered
Working together	How will the curatorial team work with the narrators?	Collaborative approach Coproduction Curator-led process Narrator-led process Participatory approach

Participants either worked alone or as part of a curatorial team, as an independent, employed by, for example, a mental health trust or charity, or unpaid. Curatorial teams were made up of people from a variety of backgrounds, such as clinical, art, or academic backgrounds and with varying degrees of lived experience of mental health service use. Some team members who were also service users stated that they felt that other team members had taken advantage of their naivety, enthusiasm, and passion, or played on their anxiety or feelings of paranoia. An example of this was with 1 participant who had worked with their former social worker and had felt unable to oppose the decision to employ this person on the curation project:

I think it’s a kind of general thing among the survivor service user community that we really want to help one another and we really want to be positive and we really want to give, to volunteer, to be involved and I think a lot of time that is taken advantage of.

#01

#### Theme 5: Ethics and Legal

The ethics and legal themes shown in Table 6 includes decisions around narrative anonymization, consent processes, data storage and security processes, project funding, payments to different parties, copyright and ownership issues, and withdrawal processes. Some participants struggled with ethical dilemmas. An example of this was a participant who considered that the withdrawal of stories may affect the collective cause of the website. They had an official policy of not allowing withdrawal; however, in certain instances, this rule was broken owing to safety concerns:

Sometimes people get cold feet or they just get nervous about stigma, and they don’t have like a real immediate threat to their livelihood or wellbeing...our official policy...we don’t take down any published content on the site at all for any reason, but realistically we do if someone has a safety concern, or there was a situation where an author became psychotic…journalism is sort of sacred...you don’t just unpublish an article because someone is upset...it’s part of the ongoing conversation...If everyone had second thoughts about their story and asked for it to be taken down, if you said ‘yes’ to all those, we would have a lot less content on the site and sometimes the stories that we do sometimes end up taking down...they were very powerful and important stories and now they are not out there, now no-one will ever read them...it's kind of like balancing the needs of the individual writers with the needs of the movement or society.

#30

**Table 6:** Ethical and legal considerations in the curation of collections.

Subthemes	Illustrative question	Identified approaches
Anonymization	How will narratives be anonymized?	Anonymize all names and details that would make anyone identifiable Anonymize narrator name and/ or details Anonymize third parties Offer each narrator choice as to what to anonymize No anonymization
Consent process	How will consent for narrative inclusion be gained?	Use of consent forms No consent process Verbal consent in person or by phone
Data storage and security	What data security procedures will be used?	Use of data encryption when storing material Use of a locked room and/or storage for data None
Funding	How will the project be funded?	Charity donation or independent funding body Crowd funding Individual sponsor Government funding Self-funded
Payments	What will be the payment processes for different parties?	Employment contract Expenses paid Fixed sum paid Royalties paid Unpaid
Copyright and ownership	Who will own the copyright to the narratives?	Curators Joint ownership Narrators Publishers
Withdrawal process	What process will be put in place for the withdrawal of narratives?	No withdrawal allowed Withdrawal permitted at any point until publication Withdrawal after publication possible

#### Theme 6: Safety and Well-Being

The safety and well-being theme shown in Table 7 includes the types of support the curators, narrators, recipients, and third parties received during the curation process, the participant’s approach to uncertainty and distress, and if and how trigger warnings were used. Participants used different approaches to deal with distress. Some participants decided to avoid the discussion of difficult subjects or to not *press* on triggering subjects such as experiences of abuse, as they considered this to be damaging. Other participants believed in tolerating the uncertainty of possible negative consequences by delving deeper into a challenging narrative and that taking risks can be beneficial to the narrators and the recipients:

Most people expect their stories to be, I don’t want to say raped, that’s not the right word, but taken advantage of for the use of other people...they were afraid that I was going to try and twist and portray their story into a harmful light or into one that would make them re-experience trauma and so I had to assure them that it was safe and that I also was not going to be damning them through my editing of their stories.

#18

Art making is risk taking...and there is benefits to the artists, the benefits then to the visitors to the public that come and really art for them is asking them questions, it is giving them different perspectives on the world, sometimes is around mental illness...art is a great space for kind of breaking down barriers between...the public and asylum and public and hospital.

#10

**Table 7:** Safety and well-being in the curation of collections.

Subthemes	Illustrative question	Identified approaches
Individual safety and well-being of the curators, narrators, recipients, and third parties	What support will be offered to the curators, narrators, recipients, and third parties?	None Online group Peer or group support Supervision Support of clinician Support of family or friend Support pack including items such as coloring books, mindfulness exercises, and support group contact details
Uncertainty and distress	How to approach uncertainty and distress?	Avoidance of difficult subjects Avoid pressing on triggers Tolerate different levels of uncertainty in times of distress and if deemed safe delve deeper
Trigger warnings	How will trigger warnings be used?	Broad trigger warning for whole collection Not used Used on all individual narratives Used on a select number of narratives

## Discussion

### Principal Findings

This study collected and analyzed the experiences of curators of collections of the lived experiences narratives of mental health service use, recovery, or madness. The resulting VOICES typology identifies 6 curatorial decision-making themes: values and motivations, organization, inclusion and exclusion, control and collaboration, ethics and legal, and safety and well-being. Our work addresses a knowledge gap related to curatorial decision making that is not available in public documents [20]. Drawing on potentially unpublished insights collected through interviews with curators, it provides a richer understanding of the intended purpose of these collections than the previous work and a richer understanding of how curators collaborated with narrators and worked to control the process of building collections. The identification of 26 subthemes and more than 100 approaches taken by the existing curators may provide the future curators with knowledge to guide their curation process.

### Relationship With Existing Work

#### Values and Motivations

It has been argued that to understand and facilitate processes of resilience and recovery, there is a need "to end the silence imposed on people with psychiatric disabilities" and value and honor the personal and collective voices of their lived experience [12]. The operationalization of the concept of recovery within mental health systems has come under increasing criticism for shifting from a collective social responsibility to a private, individual responsibility [29]. Within this general critique, the use of the recovery narrative has been criticized as positioning both the problem and its potential solution at the level of individuals [30].

Woods et al [31] posed the following question in relation to the recovery narrative: “What might be opened up, revealed, or foreclosed in telling a recovery narrative in the first-person plural?” as they argued that the recovery narrative is bound to and by the first-person singular and its efficacy is indexed to the individual. Although some participants in our study have built collections of narratives for individual purposes, others have aimed to bring together narratives united in a collective purpose or voice, to be used as a means to impact at an organizational, community, social, or political level. The curation of recovery narrative collections for a collective purpose might therefore be seen as a mechanism for addressing the criticism of the use of individual recovery narratives. Future research might evaluate the collective impact of recovery narrative collections.

#### Organization

In museums studies, the practical organizational work of curating exhibitions has been explored [32], and there is a growing interest in conceptualizing collaborative and participatory approaches, including in relation to the curation of mental health materials [33]. Research has also begun to document the complex reactions of those visiting or receiving difficult mental health materials [34]. The VOICES typology offers curation knowledge grounded in specific experiences of curating collections of narratives addressing madness, mental distress, and recovery, and hence extends this knowledge base. Further exploration of the VOICES typology is warranted as a potential decision-making tool to build collections of narratives in ways that incorporate health care values such as person-centeredness [35], inclusivity [36], and collaboration [37], and being ethically and legally sound [38] and safe [39].

#### Inclusion and Exclusion

A narrative collection brings together different voices and different narratives, and the curator holds a position of power as they make decisions on the inclusion or exclusion of different people and discourses. It has been argued that certain discourses shape and create meaning systems that gain the status of the *truth* and dominate how we define and organize both ourselves and our social world by marginalizing and subjugating alternative discourses [40]. In this instance, questions arise as to whose version of *truth* should be told in a collection and for what reason or agenda? Exclusion of narratives from a collection might contribute to a narrowing of views on how recovery can happen, potentially resulting in a disempowering discourse that acts as an antithesis to humanistic, individualized patient-centered care, which could place a greater emphasis on social and political factors in mental distress [41]. Likewise, if particular representations of recovery are considered too difficult or challenging for recipients and are therefore excluded, then that risks making certain perspectives and/or certain kinds of people invisible. Future work could evaluate the voices and messages present in particular collections and hence identify the collective message created by these collections. It might consider the opportunities and barriers to using such resources in clinical mental health practice and identify the principles for curating collections to be used for this purpose.

#### Control and Collaboration

In relation to exclusion and colonization, it has been argued that “those who have been ‘experts’ …traditionally, as researchers, academics, social and political commentators, need to think through their role. Rather than making devalued groups’ narratives merely another subject of their enquiries, or a new field under their direction, they have a chance to ‘authenticate’ service users’ experience through adding their ‘authority’, helping give it credence and legitimacy. The former represents an extension of the devaluing of outsider narratives; the latter offers the prospect of supporting their growth and empowerment” [42]. One of the core values of antioppressive practice is to give voice to and validate the experiences of oppressed people [43], and one might ask if this can be possible if their voice is filtered through a curator’s voice? Relationship parallels may be drawn between the curator and the curated and the clinician and the service user, as some participants acted with a heavy-handed editorial style, which may be viewed as oppressive, whereas others acted with the aim of not filtering the voices of the narrator through their own.

#### Ethics and Legal

A previous systematic review identified the anonymization of narratives as an ethical issue for which there is no ideal solution [20], and this study confirmed this finding, with questions raised around the best approach to take in relation to the needs of the curator, narrator, recipient, and third parties and the agenda of the collection. Curators debated the balance between their safeguarding duties and censorship and considered issues such as how to respond if the narrator was deemed too unwell to tell their story, who decides if a person has the capacity to make decisions and for what reason; what are the rights of narrators to claim their stories; and what if any protection should be given to the narrators for *their best interests* such as protecting from future stigma or prejudice in employment opportunities or relationships? These questions are pertinent in the discussion of mental health treatment around risk and safeguarding. Future work could explore parallels between the experiences of mental health treatment and the handling of the narratives of mental health treatment.

#### Safety and Well-Being

Storytelling is an act of uncertainty for the different parties involved, as what will happen during and after a narrator shares their narrative is unknown. This is especially the case where digital technologies are involved, for example, owing to the ease of access to a person’s narrative online [44]. Some approaches to health treatment, such as Open Dialogue, argue for tolerating uncertainty to enable a deeper examination of the trauma or distress in a person’s narrative [45]. These approaches state that uncertainty can only be tolerated if an individual feels safe and that safety can be established by hearing and responding to each person’s voice and point of view and legitimizing them and their narratives. The safety and well-being of the narrators, curators, recipients, and third parties was a concern for every participant interviewed. The VOICES typology highlights the participants’ curatorial approaches to uncertainty and dealing with distress, with examples of how they tried to make different parties feel safe. Future work could explore the safety and well-being of different parties after publication and the impact collections have on the lives of the narrators, curators, recipients, and third parties.

### Limitations

More participants came from the United Kingdom than any other country, which might limit the generalizability of the findings. For all collections other than one, only 1 curator per collection was interviewed, and hence only 1 perspective on the curation of each collection was collected. Interviews with multiple members of a curatorial team might reveal contrasting or even conflicting views on the process of curation.

### Conclusions

The VOICES typology identifies key decisions to consider when curating narrative collections about the lived experiences of mental health service use, recovery, or madness. The VOICES typology might be used as a theoretical basis for a good practice resource to support curators in their efforts to balance the challenges and sometimes conflicting imperatives involved in collecting, organizing, and sharing narratives. Future research might seek to document the usage of such a tool by curators and hence examine how best to use VOICES to support decision making.

## Appendix

Multimedia Appendix 1 Preliminary typology of curatorial decisions.

## Abbreviations

NEON: Narrative Experiences Online
NIHR: National Institute for Health Research
VOICES: values and motivations, organization, inclusion and exclusion, control and collaboration, ethical and legal, safety and well-being
