# Supplementary material for: The VOICES Typology of Curatorial Decisions in Narrative Collections of the Lived Experiences of Mental Health Service Use, Recovery, or Madness: Qualitative Study
Source: JMIR Ment Health. 2020 Sep 18;7(9):e16290. doi: 10.2196/16290 (PMC7532459; doi:10.2196/16290)
Supplement: Multimedia Appendix 1 [file mental_v7i9e16290_app1.pdf]

Types	Description	Considerations	Strategies
Context	How does context influence the curation of the repository?	How does the political context influence the curation of the repository?	a. Reflection on and explicit mention of political context
Purpose	What is the purpose of the repository?	What was the motivation for the collection?	a. To promote recovery in individuals experiencing mental health problems
			b. To reduce stigma
			c. To encourage people to seek help
		What is the intended impact for narrators?	a. Healing, beneficial to narrator's recovery
			b. Empowerment
		What is the intended impact for recipients?	a. Education and normalisation
			b. Promotion of hope
			c. Sharing of ideas for self-management
Curatorial Team	Who is curating the collection?	What is the composition of the curatorial team for the collection?	a. Included people skilled at collecting narratives
			b. Curatorial experience developed over time. People became expert through the curation of narratives and through developing good practice guidelines
Audience	How are the potential recipients considered?	Was there a specific population in mind?	a. Potential audience specified
		How were recipients able to interact and participate with the repository?	a. Through moderated comments
Legal and Copyright	How are legal and copyright issues dealt with?	What were the mechanisms for specifying ownership and usage of stories?	a. By providing written guidelines and agreements
			b. Through informal discussion
			c. The rights were signed over to publisher
Collection	How are narratives collected for the repository?	How was the design of collection process organised?	a. It was a structured process led by curator
		How are the narratives obtained?	a. Interviews
			b. Calls for submissions
		How are targeted types of narrator recruited?	a. GPs
			b. Hospital consultants
			c. Support groups
			d. Newsletters
			e. Advertising in the press
			f. Websites
			g. Social media
			h. Word of mouth
		How are untargeted types of narratives collected?	a. Call for submissions online
			b. Call for submissions within academic journal
		What is the process for interviewer/ interviewee preparation?	a. Discussions prior to story sharing
Selection	How are narratives selected for inclusion?	What are the mechanisms for exerting curatorial control over selection of stories?	a. Curatorial team retains tight control over choice of narrator and inclusion of narrative
			b. Curatorial team invites a broad range of submissions, and only intervenes to exclude or modify inappropriate submissions
			c. Reviewing and selecting

		What types of narratives are included?	a. Positive narratives
			b. Powerful narratives
			c. Clearly-expressed narratives
		What considerations for diversity were made?	a. Deliberately selecting a diverse range of narratives
Editing	What editing of narratives occurs?	What features of recovery narratives require specific curatorial response?	a. Discriminatory content
			b. Identification of others
			c. Presentation of distress within story
			d. Features that might be triggers
			e. Identification of the narrator
			f. Language that others might find offensive
			g. Stories that describe difficult or challenging periods in the recovery process
		What response is given to challenging features presented in narratives?	a. Request to narrator to edit narrative
			b. Curatorial team edit narrative
		What are the mechanisms for exerting curatorial control over the content of stories?	a. Curator directly shapes voice of narrative towards what might be recovery-promoting in recipient, with agreement of donor
			b. Curator and donor collaborate to shape voice of narrative towards what might be recovery-promoting in recipient
		What are the reasons for editing?	a. Editing for clarity of language
			b. Editing for meaning
			c. Editing for length
			d. Removing identifying information
Safety and Wellbeing	How are safety and well-being of narrator, recipient, and third parties ensured?	How is safety and well-being of narrator ensured?	a. Anonymization of names and identifying features
			b. Offer planning and preparation support
			c. Cultural awareness and support
			d. Provide consent agreements
			e. Support the narrator after publication with a follow up meeting or telephone call
		How is safety and well-being of recipient ensured?	a. Signpost recipient to helpline
			b. Avoid "triggering" details
		How are safety and well-being of third parties in narratives ensured?	a. Anonymization of names, identifying features
Presentation	How are the narratives presented in the repository?	What is the mode of presentation of narratives in collection?	a. Text
		What formatting restrictions apply?	a. Allowing any format;
			b. Restricting to written (and word length)
			c. Instructions relating to writing style
Language		What language is deemed acceptable?	a. Use of clinical language (with caveat)
Ordering		What order were narratives presented in?	a. Ordered based on cause of issues
			b. Ordered based on clinical diagnosis
